# Radical cystectomy with pelvic lymphadenectomy: pathologic, operative and morbidity outcomes in a Brazilian cohort

**DOI:** 10.1590/S1677-5538.IBJU.2015.0380

**Published:** 2016

**Authors:** Renato B. Corradi, Gustavo Jaime Climaco Galvão, Gabriel M. Oliveira, Vinicius F. Carneiro, Wadson Gomes Miconi, Paulo Guilherme Oliveira Salles, Walter Luiz Ribeiro Cabral, Carlos Corradi, Andre Lopes Lopes Salazar

**Affiliations:** 1 Departamento de Urologia, Intituto Mario Penna, Belo Horizonte, MG, Brasil;; 2 Departamento de Urologia, Hospital das Clínicas UFMG, Belo Horizonte, MG, Brasil

**Keywords:** Cystectomy, Lymph Node Excision, Urinary Bladder Neoplasms, Therapeutics

## Abstract

**Introduction and Objective:**

Radical cystectomy (RC) with pelvic lymph node dissection is the standard treatment for muscle invasive bladder cancer and the oncologic outcomes following it are directly related to disease pathology and surgical technique. Therefore, we sought to analyze these features in a cohort from a Brazilian tertiary oncologic center and try to identify those who could negatively impact on the disease control.

**Patients and Methods:**

We identified 128 patients submitted to radical cystectomy, for bladder cancer treatment, from January 2009 to July 2012 in one oncology tertiary referral public center (Mario Penna Institute, Belo Horizonte, Brazil). We retrospectively analyzed the findings obtained from their pathologic report and assessed the complications within 30 days of surgery.

**Results:**

We showed similar pathologic and surgical findings compared to other large series from the literature, however our patients presented with a slightly higher rate of pT4 disease. Positive surgical margins were found in 2/128 patients (1.5%). The medium number of lymph nodes dissected were 15. Major complications (Clavien 3 to 5) within 30 days of cystectomy occurred in 33/128 (25.7%) patients.

**Conclusions:**

In the management of invasive bladder cancer, efforts should focus on proper disease diagnosis and staging, and, thereafter, correct treatment based on pathologic findings. Furthermore, extended LND should be performed in all patients with RC indication. A critical analysis of our complications in a future study will help us to identify and modify some of the factors associated with surgical morbidity.

## INTRODUCTION

Radical cystectomy (RC) with pelvic lymph node dissection is the standard treatment for muscle invasive bladder cancer and a curative procedure for patients with organ confined and some with extravesical and node positive disease (1, 2). This surgical approach also provides proper disease staging based on specimen pathology and, thereafter, adjuvant treatment strategies when indicated.

Oncologic outcomes after RC are highly associated with pathologic features of the disease and surgical technique factors. Among them, extravesical disease and lymph node involvement are related to decreased overall and recurrence-free survival (2-4). Furthermore, these oncologic outcomes are also determined by the operative technique, what plays an important role in the disease control (5, 6).

The pelvic lymphadenectomy (LND) is not standardized among surgeons and institutions and its extension has been shown to be a determinant factor for improving progression-free and overall survival in patients with invasive bladder cancer submitted to RC (7). Another important point in the management of this disease is the reconstruction of the urinary tract and different urinary diversions have been described, however none of them showed superiority compared to others in terms of complications and quality of life in prospective randomized trials.

Together with the fact that muscle-invasive bladder cancer predominantly affects an elderly population, RC with pelvic LND is a major operative procedure and, therefore, is associated with an important rate of complications (8-10). The surgical morbidity impacts on hospital costs and patients survival.

Although extensively studied in different populations worldwide (1-5), bladder cancer information from Brazilian patients is lacking. Therefore, we sought to analyze the data from a cohort submitted to RC with LND for bladder cancer treatment in a Brazilian tertiary referral center and, using surgical, pathologic and morbidity parameters, compare it to the largest series from the medical literature.

## MATERIALS AND METHODS

### Patients

We identified 128 patients submitted to radical cystectomy from January 2009 to July 2012 in one oncology tertiary referral public center (Mario Penna Institute, Belo Horizonte, Brazil). In all of the cases the reason for cystectomy was bladder cancer, either invasive or non-invasive disease.

### Surgical intervention

All the patients included in the study were submitted to open radical cystectomy by 8 experienced urologic surgeons with over 5-years prior experience in urology oncologic surgeries.

Patients underwent a standard surgical procedure, including a meticulous pelvic lymphadenectomy (LND) with en bloc RC and urinary diversion. Men were submitted to prostate removal and women to hysterectomy, resection of anterior vaginal wall and bilateral salpingo-oophorectomy if those organs were present. The extent of the LND was based on surgeon’s judgment. After RC with pelvic LND, urinary diversion was performed based on preoperative and intraoperative assessments and previous patient discussion. There were not specific criteria for choosing the type of diversion, this choice was made by the surgeon and patients after discussing the risks and benefits of each type of diversion on each individual situation.

All the pathologic specimens were analyzed from the same pathologist with more than 10 years of pathology oncology experience.

### Demographic and pathologic data

Pathologic and demographic characteristics of the patient’s population were collected retrospectively from electronic medical records.

Cystectomy and lymph node specimens were evaluated according to the standard pathology protocol. Pathologic data included tumor stage and grade. AJCC TNM classification (11) was used for pathologic staging and the WHO classification (12) from 2004 for histological typing and grading.

### Post-operative complications

Complications within 30 days of surgical resection were graded using the modified Clavien classification system (13). Grade 1 and 2 complications were defined as minor, and grade 3 to 5 complications were defined as major. Regular correspondence with patients and their physician ensured that treatment received outside of our institution was accounted for in the database.

## RESULTS

The majority of the patients submitted to cystectomy in our institution were men with a median age of 67 years, ranging from 36 to 86 years and more than 90% of them presented with invasive disease in the specimen pathology; 47% with pT3-pT4 disease and 16% with pT4 tumors. Positive surgical margins were only present in 2/128 (1.5%) patients. In one of them the bladder adenocarcinoma invaded the rectal wall and in the other the high grade transitional cell carcinoma infiltrated the ileus and abdominal wall (Table-1).

**Table 1:** – Demographic, neoadjuvant chemotherapy and post operatory pathologic data, reported as absolute numbers for gender and age in years, frequency and relative number (%) for the other variables.

Male	102 (80%)
Age (range)	67 (36-86)
Neoadjuvant chemotherapy	2 (1.5%)
**Pathologic T stage**	
T0	4 (3%)
T1	8 (6%)
T2	55 (43%)
T3	40 (31%)
T4	21 (16%)
**Pathologic N stage**	
N0	99 (78%)
N1	4 (3%)
N2	24 (18%)
N3	1 (1%)
Positive margins	2 (1.5%)

Based on pre operatory histology, RC was performed because of muscular invasive disease (pT2) in most part of the cases and in 34/128 (26%) of the patients the reason for RC with LND was multiple T1 recurrence or impossibility to approach the lesion with transurethral resection. Transitional cell carcinoma was the pre cystectomy histology subtype in 117/128 (92%), squamous cell carcinoma in 7/128 (5%) and adenocarcinoma in 3/128 (3%) patients (Table-2).

**Table 2:** – Pre operatory biopsy.

pT stage	128 (100%)
pT1	34 (26%)
pT2	84 (65%)
Squamous cell carcinoma	7 (5%)
Adenocarcinoma	3 (2%)

Data reported as frequency and relative number (%)

Ileal conduit was the choice of diversion in 97/128 (75%) of the cases and orthotopic neobladder and ureterocutaneostomy was the diversion performed in 15/128 (12%) patients each (Table-3). Transitional cell carcinoma was the histologic diagnosis in the cystectomy specimen in 117/128 patients (92%) (Table-4).

**Table 3:** – Urinary diversion.

Urinary Diversion	128 (100%)
Ileal conduit	97 (75%)
Orthotopic neobladder	15 (12%)
Ureterocutaneostomy	15 (12%)
Ureterosigmoidostomy	1 (1%)

Data reported as frequency and relative number (%)

**Table 4:** – Post operative histologic subtype.

Patients submitted to RC	128 (100%)
Transitional cell carcinoma	117 (92%)
Adenocarcinoma	4 (3%)
Squamous cell carcinoma	7 (5%)

Data reported as frequency and relative number (%)

Regarding N stage, we found a 22% rate of node invasion and 86% of the patients had 10 or more nodes removed, with a median number of 15 (Table-5).

**Table 5:** – Node status and number of nodes removed.

Node Status	128 (100%)
Negative	97 (76%)
Positive	31 (24%)
Nodes removed	
Median	15
10 or more nodes removed	110 (86%)

Data reported as frequency and relative number (%)

Median length of stay (LOS) was 10 days and 21/128 (16%) patients had to be re-hospitalized within 30 days of surgery (Table-6). The most common cause was urinary infection, responsible for 13/128 (81%) events. Other reasons were dehydration, upper gastrointestinal bleeding, evisceration, deep venous thrombosis, pain requiring venous analgesics, acute renal failure, pelvic abscess.

**Table 6:** – Distribution and detailed description of length of stay, operative time, perioperative deaths, re-hospitalization and 30-day complications. Data reported as frequency and relative number (%) when applied.

Median operative time	240 min
Median length of stay	10 days
Perioperative death	0
Re-hospitalization	21 (16%)
Patients with major complications (Grade ≥3), n (%)	33 (25.7%)
**Highest grade of complications, n (%)**	
Grade 1	59 (46.1%)
Grade 2	36 (28.1%)
Grade 3	8 (6.2%)
Grade 4	14 (11%)
Grade 5	11 (8.5%)
Major complications (Grade ≥3, n) (%)	33 (25.7%)

Major complications (Clavien-dindo 3 to 5) within 30 days of cystectomy occurred in 33/128 (25.7%) patients (Table-6). Eight patients had complications classified as Clavien-dindo grade 3. In detail, the cause of re-intervention was evisceration in 4/8 (50%), pelvic abscess in 2/8 (25%), lymphocele 1/8 (12.5%) and ileal conduit ischemia in 1/8 (12.5%) patient. Among the 14/128 (11%) patients with Clavien-Dindo grade 4 complications, urinary, abdominal and pulmonary sepsis was the causes for intensive care unit (ICU) admission in 12/14 (86%) patients. The other 2/14 (14%) patients were admitted in the ICU due to myocardial infarction and hemodynamic instability with undefined cause. Re operation was needed in 8/14 (57%) of these patients and the reasons were perforated gastric ulcer 1/8 (12.5%), pyonephrosis 1/8 (12.5%), abdominal abscess 1/8 (12.5%), evisceration in 2/8 (25%) and anastomosis ileal fistula in 3/8 (37.5%) patients.

Grade 5 Clavien-Dindo complications occurred in 11/128 (8.5%) patients. The cause of death in these patients were abdominal abscess in 1/11 (9%), ureteroileal fistula in 1/11 (9%), mesenteric ischemia in 1/11 (9%), intestinal hernia 1/11 (9%), pulmonary sepsis in 1/11 (9%), urinary sepsis in 2/11 (18%), severe inflammatory response syndrome in 2/11 (18%) and cardiac events in 2/11 (18%) patients.

## DISCUSSION

Due to durable local control, long term, recurrence-free and bladder cancer specific survival there is a consensus that RC with lymph node dissection is the gold standard treatment for invasive bladder cancer. In a large series with 1054 patients, Stein et al. showed recurrence-free and overall survival at 5 years of 68% and 66%, respectively, for all patients (1), regardless of cancer stage. Other smaller series reported similar rates (2-4) and all of them showed that RC outcomes are overwhelmingly associated with the pathology, and tumor stage and lymph node status are the most important predictors for outcome (14-16). The differences in survival and recurrence rates were observed when bladder confined was compared to extravesical tumors, and intravesical muscle invasive masses showed equivalent outcomes to noninvasive ones for patients submitted to RC (1-4).

Our results show a similar rate of positive resected lymph nodes compared to cohorts from Europe (2), United States (1, 4, 17) and from a multicentric study with patients from Canadian and American centers (3). Furthermore, the rate of patients with pT3-pT4 disease reported in our current series is similar to the ones observed in the cited studies. However, if we only consider the patients classified as pT4 and compare to the contemporary cohort from Bochner et al. (17), we observe a higher percentage of patients in this pT stage in our cohort. Nevertheless, when we analyze histologic subtype, we show similar rates of transitional cell carcinoma and other bladder cancer subtypes.

Together with pathological features, surgical variables play an important role in bladder cancer outcomes and it has been shown in the SWOG-Intergroup study. In this cohort, Herr et al. (5) found substantial differences in cystectomy operative technique and patients achieved better oncological and survival outcomes when operated by urologic oncologists. Furthermore, they showed that surgical margins and number of nodes dissected were independent predictors of local recurrence and survival (5). Indeed, positive surgical margins increase the metastatic progression risk and adversely affect cancer-specific survival (18). We observed positive margins in less than 2% of our cases, finding that is in accordance with the ones from other series (5, 19) and in part reflects the surgical expertise of the surgeons from our institution.

Although the number of pelvic nodes varies widely between individuals (20) it is associated to the extent of the lymph node dissection among experienced surgeons (21). Knowing that, the SWOG researchers analyzed the number of lymph nodes dissected and its importance for disease control, showing better outcomes for patients with more than 10 nodes removed. Also, other series measured the extension and quality of the lymphadenectomy (LND) by defining a quantity of nodes that should be dissected, and this minimum number ranged from 10 to 16 (15, 22-24). In our cohort, we report a 86% rate of 10 or more and a median of 15 nodes removed, acceptable numbers when we take into account the cutoff number from the SWOG group (5).

The boundaries of extensive LND for bladder cancer include the ones from the limited LND and the cephalad dissection extended to the crossing of the ureters with common iliac arteries and tissue along the lateral and medial portion of internal vessels (25). This LDN approach was important for increasing the survival in node positive and negative patient groups. In the Stein et al. series, despite cancer node involvement, 31% and 23% of the patients were alive 5 and 10 years after RC with extensive LND, respectively. For patients with node negative disease, it also showed a positive effect on survival, suggesting a role in removing occult microscopic metastasis (15, 26, 27). Furthermore, in a retrospective study comparing limited to extended LND, the second approach was responsible for increased overall and recurrence-free survival (7). Therefore, since higher lymph node yield impacts positively in overall and disease-specific survival (6, 28), efforts must be made to its widespread adoption in our and other centers worldwide.

Another important step in the surgical management of invasive bladder cancer is the reconstruction of the urinary tract either with orthotopic or heterotopic, continent or incontinent urinary diversions. The surgical morbidity following them is significant (29) and has not been associated with the type of diversion (30). Orthotopic neobladder provides the patient with the potential for normal voiding function and continence together with superior cosmetic appearance compared to heterotopic conduits. Nevertheless, randomized prospective trials using appropriate quality of life instruments to compare the different types of diversions are lacking. In our institution, the ileal conduit was used in the vast majority of the cases and its choice was driven by patient option and surgeon experience.

Prior series reported early complications, defined as the ones affecting patients within 30 days of surgery, and their rate ranged from 20-57% (10). In a study from our colleagues from São Paulo, Brazil, Meller et al. (8) showed a rate of immediate and late complications of 19% and the group from the University of Michigan (9), in a study with 2.538 patients, reported at least 1 complication in 774 (30.5%) patients. A common characteristic of these cited studies and others from the literature is the lack of a formal complication reporting system. Without this, it is difficult to compare data from different studies and the morbidity of the procedure may be underestimated. Therefore, we report our complications with a well defined and accepted reporting method, the Clavien-Dindo classification (13).

Compared to a large series with 1142 patients from Shabsig et al. (10), we found a higher rate of major complications, defined as Clavien-Dindo grade ≥3 within 30 days. In a multivariable analysis from this same series (10), it has been shown that advanced age, prior abdominal/pelvic surgery, blood loss and ASA score >2 were significant predictors for complications. In our cohort, different types of complications were observed and identifying the factors that are associated with them should be the aim of a future study. This will help us improve our institution quality of care and will also be important for proper patient counseling.

This is a retrospective descriptive analysis of the surgical and pathologic outcomes from a contemporary Brazilian cohort submitted to RC and pelvic LND. The short follow-up did not allow us to make a meaningful oncologic and survival analysis of our data, and we believe this is the greatest limitation of our study.

## CONCLUSIONS

Survival and oncologic outcomes following RC are associated with pathologic stage and surgical variables. Therefore, efforts should focus on proper disease diagnosis and staging, and, thereafter, correct treatment based on pathologic findings. Furthermore, extended LND should be performed in all patients with RC indication. A critical analysis of our complications and their correlation with patient’s characteristics, pre, per and post-operative management plans will help us to identify and modify some of factors associated with surgical morbidity. This should be the aim of a future study in our institution.
